# Time-averaged disease activity fits better joint destruction in rheumatoid arthritis

**DOI:** 10.1038/s41598-017-05581-w

**Published:** 2017-07-19

**Authors:** Hideaki Tsuji, Koichiro Yano, Moritoshi Furu, Noriyuki Yamakawa, Katsunori Ikari, Motomu Hashimoto, Hiromu Ito, Takao Fujii, Wataru Yamamoto, Koichiro Ohmura, Atsuo Taniguchi, Shigeki Momohara, Fumihiko Matsuda, Cornelia F. Allaart, Hisashi Yamanaka, Tsuneyo Mimori, Chikashi Terao

**Affiliations:** 1 Department of Rheumatology and Clinical Immunology, Kyoto University Graduate School of Medicine, Shogoin Kawaharacho 54, Sakyo-ku, 606-8507 Kyoto Japan; 2 Institute of Rheumatology, Tokyo Women’s Medical University, 8-1, Kawada-cho, Sinjuku, 162-8666 Tokyo Japan; 3 Department of the Control for Rheumatic Diseases, Kyoto University Graduate School of Medicine, Shogoin Kawaharacho 54, Sakyo-ku, 606-8507 Kyoto Japan; 4 Department of Immunology and Rheumatology, Kyoto Katsura Hospital, 17-banchi, Yamada Hirao-cho, Nishikyo-ku, 615-8256 Kyoto Japan; 5 Department of Orthopedic Surgery, Kyoto University Graduate School of Medicine, Shogoin Kawaharacho 54, Sakyo-ku, 606-8507 Kyoto Japan; 6 Department of Health Information Management, Kurashiki Sweet Hospital, Nakasho, 3542-1 Kurashiki Japan; 7 Department of Center for Genomic Medicine, Kyoto University Graduate School of Medicine, Shogoin Kawaharacho 54, Sakyo-ku, 606-8507 Kyoto Japan; 8 Leiden University Medical Center, Albinusdreef 2, Leiden, 2333 ZA The Netherlands; 9 Center for the Promotion of Interdisciplinary Education and Research, Kyoto University, Yoshida-honmachi, Sakyo-ku, Kyoto, 606-8501 Japan; 10 Division of Rheumatology, Immunology, and Allergy, Brigham and Women’s Hospital, Harvard Medical School, 75 Francis Street, Boston, MA 02115 USA; 11 Division of Genetics, Brigham and Women’s Hospital, Harvard Medical School, 75 Francis Street, Boston, MA 02115 USA; 12 Program in Medical and Population Genetics, Broad Institute, 415 Main Street, Cambridge, MA 02142 USA

## Abstract

Disease activity of rheumatoid arthritis (RA), evaluated as Disease Activity Score (DAS), is associated with joint destruction. Since joint destruction reflects the history of disease activities, we hypothesized that time-averaged disease activity would better correlate with joint destruction than one-time disease activity. We recruited RA patients in IORRA (n = 557) and KURAMA (n = 204) cohorts, and calculated time-averaged DAS28 to model a modified Sharp/van der Heijde score (SHS). We evaluated the fitting of the model using time-averaged DAS28 among 1000 models in which we randomly picked up one-time DAS28. We also used clinical disease activity index (CDAI) or data in the BeSt study (European population). After conditioning on autoantibody and disease duration, time-averaged DAS28 showed significant improvement of model fitting compared with one-time DAS28 in both cohorts (p = 0.001 and 0.034, respectively). Time-averaged CDAI also showed a better fit. Integration of multiple DAS fit SHS better in the BeSt study. A good fit of time-averaged DAS could be observed using five to six time points of DAS. In conclusion, time-averaged disease activity fits the joint destruction model better than one-time disease activity. Usage of time-averaged disease activity as a covariate would increase the power of studies to identify novel correlates of joint destruction.

## Introduction

Rheumatoid arthritis (RA) is a systemic disease of chronic synovitis and leads to joint destruction^1^. Treatment with disease modified anti-rheumatic drugs (DMARDs) and biologics improve disease activities of RA, but even during the treatment, inflammatory joint damage such as erosions and joint space narrowing can be progressive and irreversible due to the remaining disease activity, and result in functional impairment^2^. Investigation of risk factors associated with joint destruction of RA is important for its prevention. Evaluation by conventional plain radiographs of hands and feet using modified Sharp/van der Heijde score (SHS) is a standard imaging technique for the assessment of joint damage in patients with RA^3, 4^.

Previous studies proved that high disease activity, commonly evaluated by Disease Activity Score 28 (DAS28), presence of cyclic citrullinated peptides antibodies (CCP) and rheumatoid factor (RF), disease duration, and genetic components correlate with joint destruction in RA^5–12^. CCP is an antibody which recognizes citrullinated peptides including filaggrin^13^, vimentin^14^, fibrin^15^, a-enolase^16^ and so on. Presence of CCP and RF are closely associated with each other, and 50% of RA patients have both CCP and RF^11^. We previously showed that RF is correlated with joint destruction independently of CCP, as CCP-negative RF-positive RA showed more joint destruction than CCP-negative RF-negative subjects^17^.

Since the prolonged period of inflammation leads to joint destruction, the association between DAS and SHS is reasonable. Most previous studies used one-time DAS28 as well as disease duration as independent variables for the assessment of the correlates of joint destruction^18, 19^. However, there remained a question whether one-time DAS is the best correlate for the assessment, since radiographic changes were considered to reflect the cumulative history of the disease activities, which changes over the disease period as well as the treatment course of RA.

On this point, the mathematical assessment of radiographic progression over time had been argued in previous studies. Some studies used not one-time DAS28 but time-integrated values of disease activities calculated as the area under the curve (AUC DAS) or generalized estimating equations (GEE), and the results correlated with radiographic progression in the cohorts of RA patients^8, 18–20^. All these observations suggest the appropriation of a linear model for radiographic progression based on the curve fitting of longitudinal data. However, to the best of our knowledge, there are no analyses to compare time-integrated values of DAS with one-time DAS to show superiority of time-integrated values. Furthermore, there is no information about how many DAS are necessary to get the better fit on joint destruction.

Here, we calculated time-averaged disease activity by using the available consecutive disease activity data and evaluated the superiority of time-averaged disease activity to one-time disease activity to fit the model for the assessment of joint destruction.

## Results

**Figure 1:**
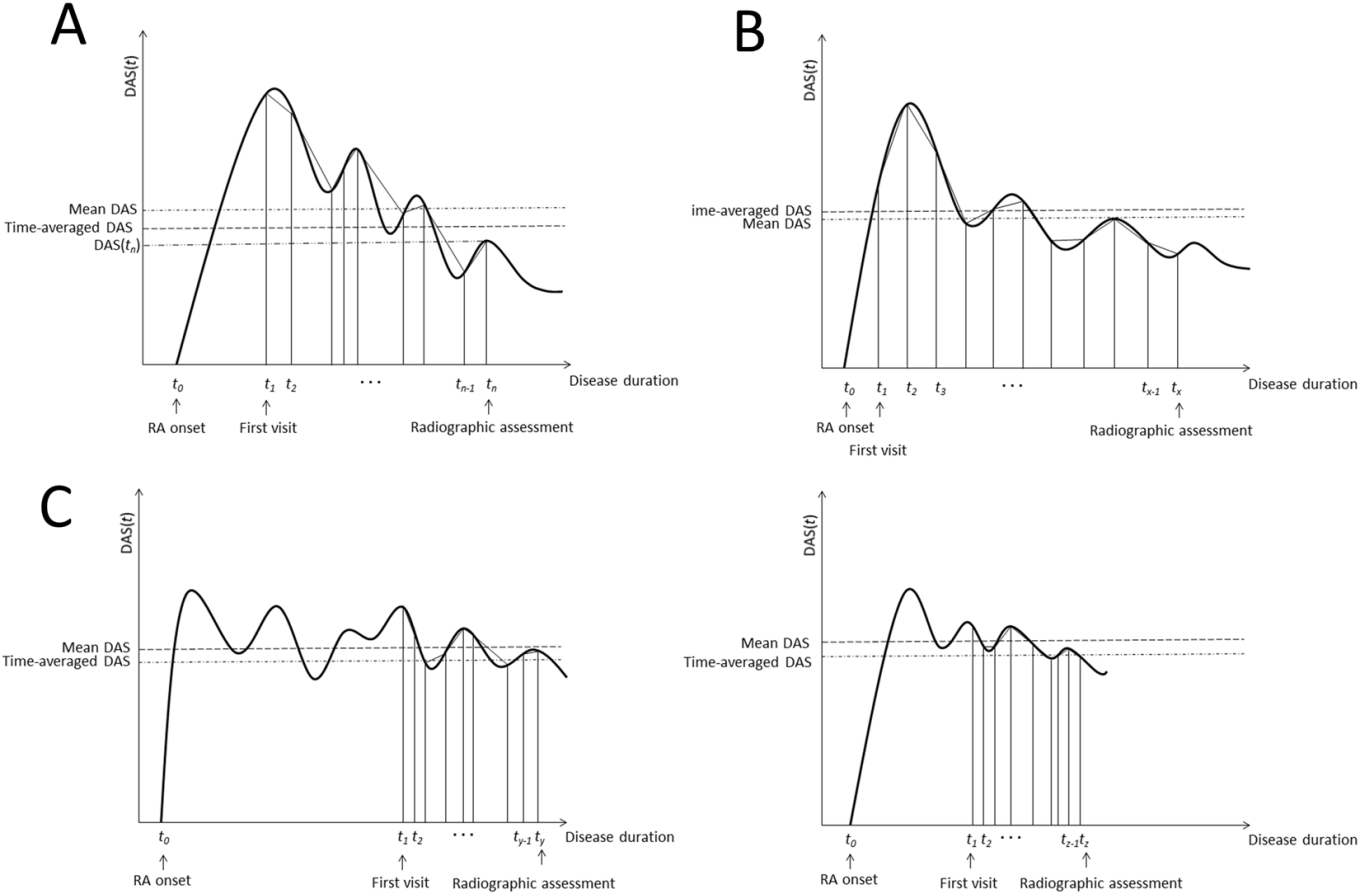
A new model to fit joint destruction in RA. (**A**) Schematic image of calculation of time-averaged DAS. We performed integration of DAS from the first visit to the hospitals to the time for the assessment of SHS, and divided the value by the time. (**B**) Schematic image of ideal cases who start to come to hospital soon after developing RA and are periodically followed-up. (**C**) Schematic image of cases who start to be followed-up later after development of RA.

We hypothesized that product of time-averaged disease activity and disease duration could well approximate cumulative disease activity (Fig. 1A) especially when patients started to come to the hospital soon after RA onset and received periodical follow-up (Fig. 1B). In such cases, time-averaged disease activity might fit joint destruction in RA very well. We also assumed that even when patients started to be followed-up in hospital several years after RA onset and/or they did not have so many time points of evaluation of disease activity (Fig. 1C), time-averaged disease activity using multiple time points would better correlate with joint destruction than one-time.

We used data from two different cohorts in Japan, the IORRA cohort (1^st^ set, n = 557) and the KURAMA cohort (2^nd^ set, n = 204), for evaluation of this hypothesis. Detailed characteristics of the two sets are shown in Table 1. Since the IORRA cohort had joint X-rays around five years after disease onset with periodical disease activity data, this cohort is similar to the model in Fig. 1B. Since the KURAMA cohort had joint X-rays with variable disease duration and data of many time points of disease activity, this serves to address the model in Fig. 1C.

**Table 1:** Baseline characteristics of patients for the assessment of time-averaged DAS28 in the Japanese cohorts.

	1^st^ set (IORRA)	2^nd^ set (KURAMA)
Number of subjects	557	204
Age, years	55.2 ± 12.4	61.7 ± 12.8
Female sex	85%	85%
Stage	Na	2.53 ± 1.08
Disease duration, years	4.72 ± 1.01	8.08 ± 6.08
Observation time, days	910 ± 465	239 ± 103
Number of measurement, times	5.76 ± 2.53 (1–12)	6.13 ± 3.34 (2–19)
SHS(total)	Na	73.8 ± 80.8
SHS(hand)	22.7 ± 23.4	50.2 ± 55.2
DAS28(ESR)	3.56 ± 1.31	3.22 ± 1.34
DAS28(CRP)	Na	2.39 ± 1.06
CDAI	9.33 ± 8.12	9.02 ± 7.86
First DAS28(ESR)	4.23 ± 1.26	3.60 ± 1.46
First DAS28(CRP)	Na	2.77 ± 1.18
First CDAI	13.29 ± 8.84	11.7 ± 9.32
Latest DAS28(ESR)	3.30 ± 1.20	3.05 ± 1.19
Latest DAS28(CRP)	Na	2.10 ± 0.93
Latest CDAI	7.64 ± 6.68	7.13 ± 6.25
Time-averaged DAS28(ESR)	3.60 ± 1.05	3.18 ± 1.10
Time-averaged DAS28(CRP)	Na	2.33 ± 0.81
Time-averaged CDAI	9.50 ± 6.35	8.47 ± 5.62
RF positive	87%	83%
CCP positive	85%	81%

We calculated time-averaged DAS28 in each patient based on the methods illustrated in materials and methods and Fig. 1A. Mean and standard deviation of the time-averaged DAS28 seemed comparable to those in one-time DAS28 (Table 1), indicating difference observed between one-time and time-averaged DAS28 could not be explained by difference in score of DAS28 itself.

**Figure 2:**
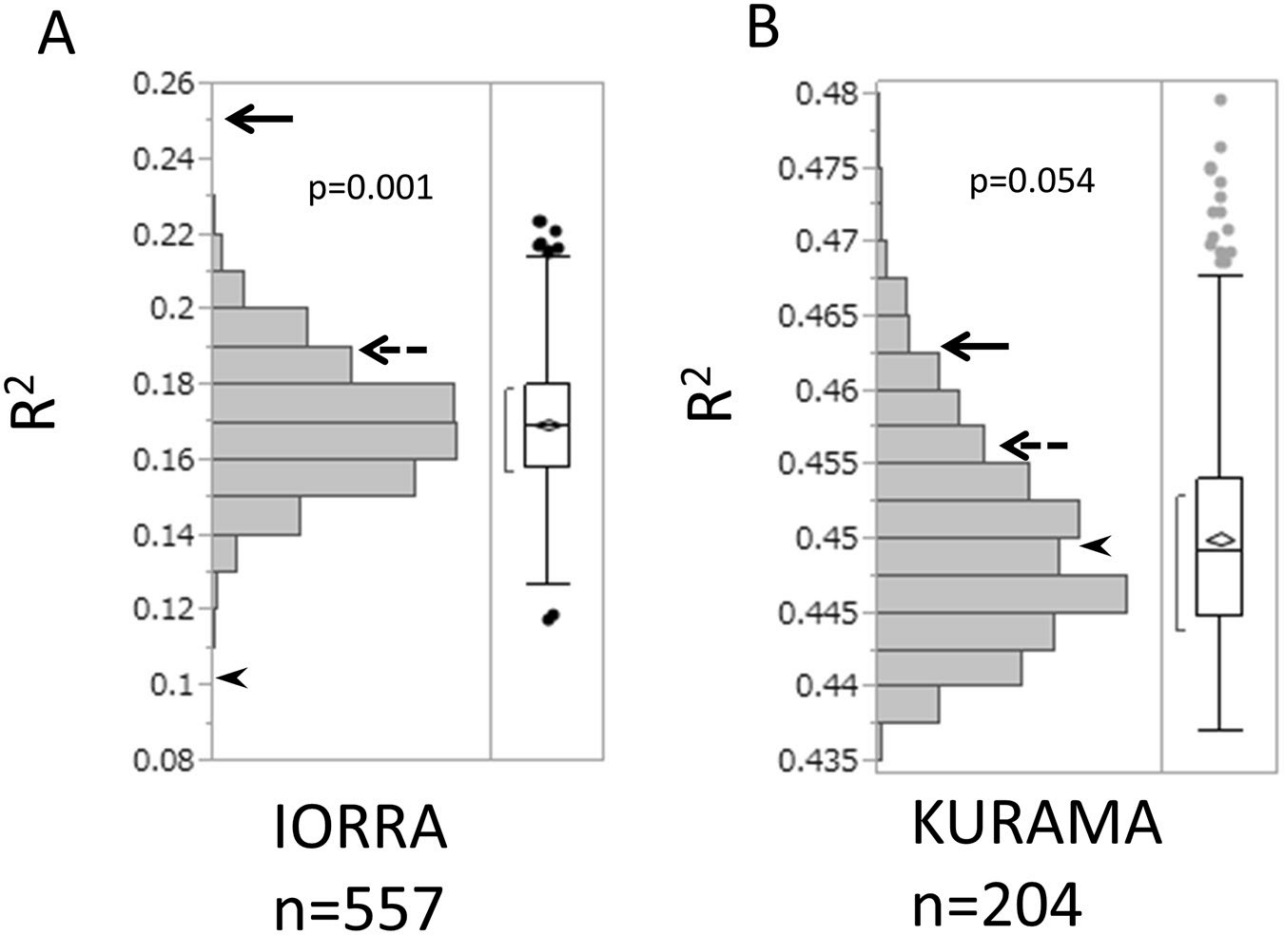
Better fit of time-averaged disease activity on joint destruction than one-time disease activity in the Japanese subjects. Empirical p-values of time-averaged DAS28 are indicated for (**A**) the IORRA and (**B**) KURAMA cohorts. Bar charts indicate distribution of R^2^ of the 1000 one-time DAS28. The solid and broken arrows indicate the time-averaged DAS28 and the latest DAS28, respectively. The arrowheads indicate the first DAS28.

We first performed linear regression analysis with time-averaged DAS28 as an independent variable using RF and disease duration as covariates in the 1^st^ set. We obtained R squared value (R^2^) of the model and compared it with those of the 1000 models using one-time DAS28 (for details, see Materials and Methods). As a result, we found superiority of the time-averaged DAS28 over the one-time DAS28 (p = 0.001, Fig. 2A and Supplementary Table 1).

Next, we analyzed data in the 2^nd^ set in which dense data of disease activity were available for a short period of time in spite of long disease duration. As a result, the 204 samples revealed better fit in the model with time-averaged DAS28 than those with one-time DAS28. (p = 0.034, Supplementary Figure 1 and Supplementary Table 2) When we extracted hand SHS data and conducted the same analysis, we obtained comparable results of superiority of time-averaged DAS28 to one-time DAS28 (p = 0.054, Fig. 2B, and Supplementary Table 1).

Next, we used CDAI as an independent variable instead of DAS28 to confirm that our model was robust. Time-averaged CDAI tended to fit better than one-time CDAI in both sets 1 and 2 (Supplementary Tables 1 and 2). We further confirmed that the model by using time-averaged DAS28 consistently tended to fit better than one-time DAS28 where we used log-transformed SHS as alternative dependent variables or used DAS28 (CRP) or log-transformed disease duration as alternative independent variables (Supplementary Tables 1 and 2).

When we used one-time DAS28 evaluated at the first visit or at the time nearest the time of joint X-rays instead of randomly selected one-time DAS28, the DAS28 did not show very good fit consistently across the 1^st^ and 2^nd^ sets (Fig. 2A and B and Supplementary Tables 1 and 2). Since the IORRA cohort evaluated disease activity every six months, the disease activities for some patients were evaluated at a date far from the date of joint X-rays. However, when we selected the 516 patients whose DAS 28 data were evaluated within six months before joint X-rays, the last DAS 28 again showed a poor fit (Supplementary Figure 2), indicating that disease activity at last visit before or the same time of X-ray does not fit joint destruction very well.

**Figure 3:**
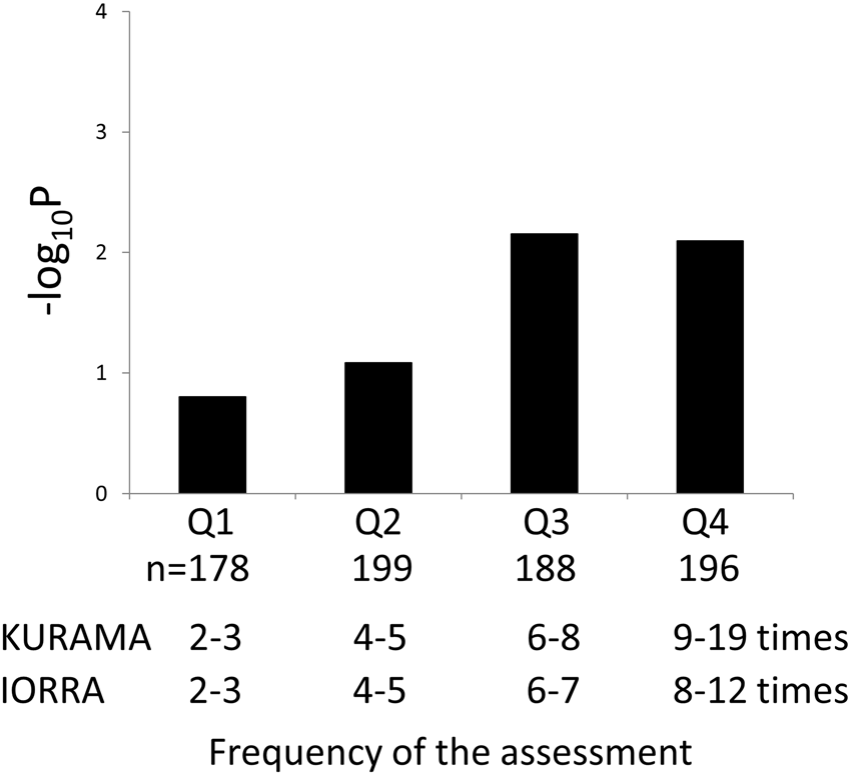
Good fit of time-averaged DAS28 was driven by subjects with more numbers of DAS28. The bar charts indicate −log_10_ empirical P-values of R^2^ of time-averaged DAS28 in each quadrant based on the number of time points of DAS28.

When we divided the cohorts according to the number of assessment of DAS28, linear model using time-averaged DAS28 tended to fit well in a subgroup with more time points of DAS28 (Fig. 3). While difference in the characteristics of the two cohorts seemed to result in slightly different tendencies among the quadrants, a substantially better fit was observed in both cohorts in the quadrant with more than six evaluations (Supplementary Figure 3).

**Figure 4:**
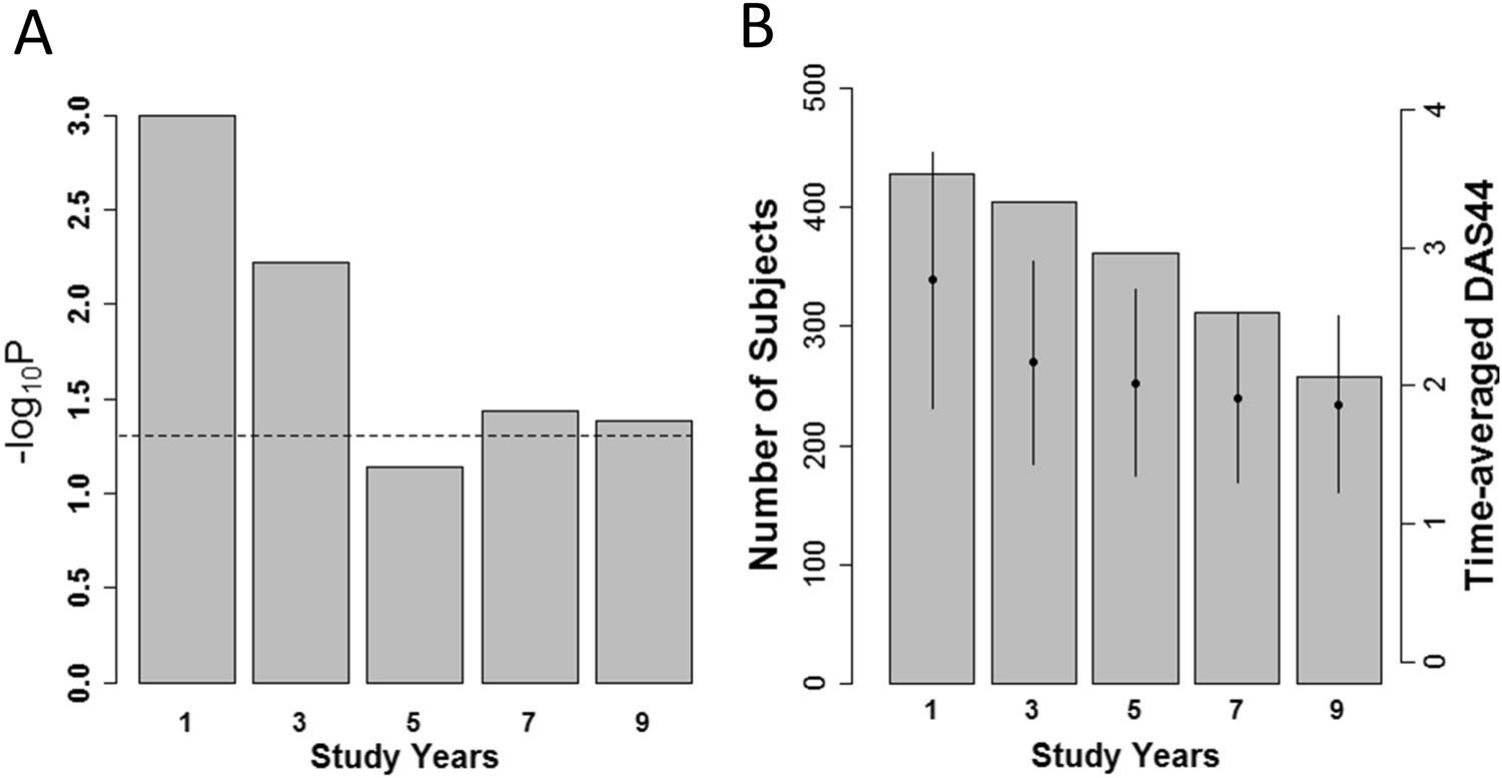
Better fit of time-averaged disease activity on joint destruction than one-time disease activity in the BeSt study. (**A**) Empirical p-values of time-averaged DAS44 from the registry to the study years are indicated. The horizontal line indicates p-value of 0.05. (**B**) Bar charts indicate the number of the subjects in the study years. Dot plots and error bars indicate mean and standard deviation of DAS44 in the study years. IORRA = Institute of Rheumatology, Rheumatoid Arthritis; KURAMA = Kyoto University Rheumatoid Arthritis Management Alliance; na = not applicable; SHS = modified Sharp/van der Heijde score; DAS28 = disease activity score 28; CDAI = Clinical Disease Activity Index; RF = rheumatoid factor, and CCP = anti-cyclic citrullinated peptide antibody. The data were expressed with mean ± standard deviation for variables.

Lastly, we analyzed data of the BeSt study to confirm that the current findings are observed in a European cohort with definitive treatment and stringent control of disease activity. The characteristics of the subjects are shown in Table 2. As a result, we found that time-averaged DAS44 showed a substantially better fit than one-time DAS44 across the different years evaluated (Fig. 4A). We observed this better fit even in the first year, indicating that five time points of DAS might be enough for the better fit. This seemed to be compatible with the results in the Japanese data. We also confirmed that DAS44 at the first point or at the time nearest to the X-ray evaluations did not show good fits (data not shown). The decrease of the number of subjects and low disease activity in the later follow-up years of the study seemed to explain the better p-values in the first and the third year than years after the five years (Fig. 4B).

**Table 2:** Characteristics of subjects in the BeSt study.

	BeSt study
Number of subjects at registry	508
Age, years	54.4 ± 13.2
Female sex	67.5%
RF positive	64.5%
CCP positive	61.5%
ΔSHS (in the first year)	2.09 ± 7.91
First DAS44	4.45 ± 0.87
Latest DAS44 (in the first year)	2.14 ± 1.05
Time-averaged DAS44 (in the first year)	2.77 ± 0.92

Collectively, these results suggest that a model of joint destruction in patients with RA using time-averaged disease activity as an independent variable fits better than a model using one-time disease activity and has better power to identify unknown correlates of joint destruction.

## Discussion

In this study, we showed the superiority of time-averaged disease activity to one-time disease activity to assess association with joint destruction using three independent cohorts. We also compared time-averaged disease activity with the first or latest disease activity, since these values were representatives of disease activity frequently used in the previous studies to fit joint destruction.

Although previous studies argued the mathematical assessment of joint destruction^8, 18–22^, there were no analyses that compared time-integrated values with one-time disease activity.

While the two Japanese cohorts of SHS did not have inter-observer coefficients, high intra-observer coefficients as well as the results from our previous studies using the data support the accuracy of the SHS^6, 23^. We observed the better fit using time-averaged disease activity in the two Japanese cohorts with different characteristics of observation period and density of disease activity data. We also confirmed the findings using a European cohort with definitive treatment and stringent control of disease activity. Among the three cohorts, the BeSt study is the most similar to the model shown in Fig. 1B. Thus, we can generalize our model in a wide variety of RA data regardless of observation period, frequencies of disease activity data and populations. Since we showed better fit using time-averaged CDAI, we can apply this model into data sets or cohorts without consecutive laboratory data. Comparable results of SHS between data of only hands and that of hands and feet suggest that we can apply this model even when SHS data of only hands are available.

Since the IORRA cohort has data of SHS in patients with RA with disease duration around 5 years^24^, they showed shorter disease duration with small variance, smaller SHS and younger age. These differences in cohorts could also explain the difference in R^2^ between the two cohorts in the Japanese.

We used RF as a covariate since we previously showed high co-occurrence of RF and CCP and a joint-destructive association of RF which was independent of CCP. When we used CCP as a covariate or CCP and RF as covariates, we obtained similar results (data not shown).

We assumed that treatment effects were substantially reflected in disease activity. In addition, the validation using the BeSt study supports the current findings.

We also showed that the better fit on joint destruction could be obtained using disease activity from five to six time points. Since the BeSt study had a limited number of subjects with less than four time points of DAS, we could not subdivide the patients as in the Japanese subjects.

It is interesting that the better fit did not appear to be completely dependent on number of time-points in a definitive meaning. The better fit seemed to be obtained using five to six time-points and no further improvements were obtained in the subjects with more time-points in the three cohorts.

The results in the BeSt study also suggest that better fit might be brought about by high to moderate disease activity during disease course since we did not find improvement after the third year. This seems reasonable since high disease activity leads to joint destruction and long period of remission would make the average of disease activity difficult to properly reflect high disease activity in a short period.

Our model is an integration of disease activity from the first visit to the time of radiographic assessment approximating average of disease activity during disease course of the patients. This model is intuitive and it should be noted that while time-averaged disease activity seemed comparable to one-time disease activity as a whole in the Japanese cohorts, we obtained significant results, indicating the importance of using appropriate disease activity in each patient to model SHS.

Our model has limitations as well: we cannot evaluate the disease activity between onset of RA and the first visit to hospitals since there are no measures to obtain the patient’s DAS of that period. Time-averaged disease activity using a limited number of time points may not very accurately approximate disease activity over the disease course. In addition, we could not capture variation of DAS between the consecutive visits if DAS fluctuated during the two visits. As we observed in the BeSt study, long term of low disease activity or remission would confound fit of the model since time-averaged disease activity cannot fully project short period of high disease activity and slight difference in disease activity in remission between patients do not seem to well correlate with difference in joint destruction. We excluded patients whose disease durations were over 20 years because SHS do not linearly correlate with DAS in long disease duration^25^. Weighing DAS based on disease duration would lead to better fit of this model. The time-averaged DAS is cumulative disease activity over an observation period estimated by joint swellings, tendencies, and CRP (or ESR). Thus, disease activity not evaluated by these components can be missed and integration of these factors would improve the fitting. In addition, usage of imaging modalities including micro CT which may evaluate joint destruction more accurately than X-rays would lead to better fitting of this model.

In spite of these limitations, the highly significant results in the current study indicate the importance of using disease activity by integrating multiple time points to fit SHS. It would be interesting to perform linear regression analysis using this model to find yet-to-be-identified correlates of joint destruction in RA. In addition, it would be interesting to use the current model to show associations between genetic components or autoantibodies and joint destruction which are independent of disease activity.

Intensive treatment such as biological DMARDs can be used in the patient from the early stages of RA and a subset of patients show repair of joint destruction after such treatment. Since we do not have enough number of sequential SHS data in patients treated with biological DMARDs, future studies would clarify whether healing effect of biological DMARDs can be appropriately modeled by our model with some modifications.

In summary, we showed that integration of time-averaged disease activity in the linear regression model fits SHS better than one-time disease activity. Time-averaged disease activity is a simple mathematical model and its use as a covariate would increase the power of studies to identify novel correlates of SHS.

## Materials and Methods

### Japanese Patients

We recruited 557 patients in the Institute Of Rheumatology, Rheumatoid Arthritis (IORRA) cohort^26^ and 204 patients in the Kyoto University Rheumatoid Arthritis Management Alliance (KURAMA) cohort^6^, whose data of SHS, DAS28, CDAI (Clinical Disease Activity Index), RF, CCP, and disease duration were available. DAS28/CDAI at regular six-month intervals was available in the IORRA cohort since this cohort systematically collects clinical information every six months.

DAS28/CDAI was available for every hospital visit in the KURAMA cohort since this cohort aims to deeply phenotype registrants to get insight of the disease. Since the KURAMA cohort was launched in 2011, the period of which DAS28 or CDAI are available is shorter than the disease duration. As SHS may not linearly reflect disease duration among patients with long RA history^25^, we did not recruit patients with more than 20 years duration of RA in the KURAMA cohort.

Written informed consent to use clinical data was obtained from all the participants. This study was conducted in accordance with the Declaration of Helsinki and its amendments and was approved by the Ethics Committee in Tokyo Women’s Medical University Hospital and Kyoto University Hospital.

### SHS score

X-rays of the patients’ joints were assessed by the SHS method^3, 4^. The X-rays in the IORRA cohort were taken between 4 to 6 years after onset of RA, while the timings of X-rays in the KURAMA cohort were variable. The X-rays were blindly evaluated by trained rheumatologists (KY and MF for the IORRA and KURAMA, respectively) with intra-observer agreement of 0.93 and 0.95, respectively (details omitted)^6^. Since foot X-rays were not available for patients in the IORRA cohort, the scores for the hand X-rays were used for analyses and shown in the main figures. Scores for hand and foot were analyzed only in the KURAMA cohort and shown in Supplementary materials.

### Clinical laboratory variables as covariates

The baseline characteristics of the RA patients were obtained as covariates from the database of the IORRA and KURAMA cohorts, including disease duration, RF (by latex turbidimetric immunoassay, positive at >20 IU/mL), and CCP (Medical and Biological Laboratories, by chemiluminescent enzyme immunoassay, positive at ≥4.5 U/mL). These variables were decided based on the previous studies^5–12^.

### The BeSt study

To validate the findings in the two Japanese cohorts, we used the data in the BeSt study, in which 507 subjects with early RA were registered to be treated by finely-defined treatment regimen to control disease activity^27^. The SHS inter-observer correlation coefficient was 0.96 in this study, as previously described^27^. Patients visited medical institutions every 3 months and their disease activities were evaluated as DAS44 on a regular basis. X-rays for hands and feet were taken annually. The data including SHS, DAS44 and basic clinical information was provided by one of the authors (CAF). This study was approved by the medical ethics committees and boards of all participating hospitals. Written and oral information about the trial, including an explanation about using the data (under code) for research on RA in future studies was obtained by the patients’ rheumatologists.

### Calculation of time-averaged disease activity

We calculated time-averaged DAS28 as follows to model SHS together with the other covariates. Time-averaged DAS is the cumulative effect of disease activity estimated by joint swellings, tendencies, and CRP (or ESR). The following equation was used to approximate average DAS from onset of RA to the time for assessment of SHS:

$${\int }_{0}^{n}DAS(t)dt\approx [\sum _{k=2}^{n}[DAS28({t}_{k})+DAS28({t}_{k-1})]\times ({t}_{k}-{t}_{k-1})]/2({t}_{n}-{t}_{1})$$

where n indicates the number of DAS measurement from the first visit to the time of the radiographical assessment, t_k_ indicates the time of the k-th measurement of DAS from the first visit, and DAS(t_k_) indicates the DAS score of k-th measurement. We also applied the same statistical framework to CDAI in the Japanese cohorts and to DAS44 in the BeSt study.

### Calculation of random disease activity

We randomly picked up disease activity 1000 times in each patient from multiple disease activities of different visits during the observation periods, and constructed 1000 sets of one-time disease activity in the RA patients.

### Statistical Analysis

In the Japanese cohorts, linear regression analysis was performed with SHS as a dependent variable and positivity of autoantibodies (RF), disease duration, and one-time or time-averaged disease activity (DAS28 or CDAI) as independent variables. We used DAS28 (ESR) for the analysis and confirmed the results by using DAS28 (CRP) which were available only in the KURAMA cohort. We also performed linear regression analysis with log-transformed SHS score (log_10_(SHS + 1)) and log-transformed disease duration as a dependent variable and an independent variable, respectively.

In the BeSt study, the difference in SHS (ΔSHS) between the evaluation year and the time of registration was calculated and used as the dependent variable. Since we found a part of the samples showed extreme values of ΔSHS resulting in a deviated distribution, we log-transformed ΔSHS (log_10_(ΔSHS + 1)). We used RF positivity, indicator variables of treatment arms and one-time or time-averaged DAS44 as independent variables.

We performed linear regression analysis with use of each of the 1000 one-time disease activity or time-averaged disease activity. We evaluated fitting the model of time-averaged disease activity by comparing R^2^ of the linear regression model with those of the 1000 models using one-time disease activity. We also evaluated fitness of the model with use of disease activity at the first visit and the latest visit before or at the same time of joint X-rays.

We also divided the subjects into four groups based on the number of available time points of disease activity in the Japanese cohorts to analyze time-points-dependency of model fitting.

A p-value less than 0.05 was set as the cut-off level for statistical significance. Statistical analyses were performed using R software or JMP Pro 11 software.

## Electronic supplementary material

Supplementary information
