# Supplementary material for: Time-averaged disease activity fits better joint destruction in rheumatoid arthritis
Source: Sci Rep. 2017 Jul 19;7:5856. doi: 10.1038/s41598-017-05581-w (PMC5517434; doi:10.1038/s41598-017-05581-w)
Supplement: Supplementary file 1 — Supplementary information [file 41598_2017_5581_MOESM1_ESM.doc]

Supplementary information

Time-averaged disease activity fits better joint destruction in rheumatoid arthritis.

Hideaki Tsuji, MD^1)^, Koichiro Yano, MD, PhD^2)^, Moritoshi Furu, MD, PhD^3)^, Noriyuki Yamakawa, MD, PhD ^1), 4)^, Katsunori Ikari, MD, PhD ^2)^, Motomu Hashimoto, MD, PhD ^3)^, Hiromu Ito, MD, PhD ^5)^, Takao Fujii, MD, PhD ^3)^, Wataru Yamamoto^6)^, Koichiro Ohmura, MD, PhD ^1)^, Atsuo Taniguchi, MD, PhD ^2)^, Shigeki Momohara, MD, PhD ^2)^, Fumihiko Matsuda, PhD ^7)^, Cornelia F. Allaart, MD, PhD^8)^, Hisashi Yamanaka, MD, PhD ^2)^, Tsuneyo Mimori, MD, PhD ^1)^, Chikashi Terao, MD, PhD ^7), 9), 10), 11),12)^

^1^Department of Rheumatology and Clinical Immunology; ^3^Department of the Control for Rheumatic Diseases; ^5^Department of Orthopedic Surgery, ^7^Department of Center for Genomic Medicine; ^9^Center for the Promotion of Interdisciplinary Education and Research, Kyoto University Graduate School of Medicine, Shogoin Kawaharacho 54, Sakyo-ku, Kyoto, Japan; ^2^Institute of Rheumatology, Tokyo Women’s Medical University, 8-1,Kawada-cho,Sinjuku,Tokyo, Japan; ^4^Department of Immunology and Rheumatology, Kyoto Katsura Hospital, 17-banchi, Yamada Hirao-cho, Nishikyo-ku, Kyoto, Japan; ^6^Department of Health Information Management, Kurashiki Sweet Hospital, Nakasho 3542-1, Kurashiki, Japan; ^8^Leiden University Medical Center, Leiden, The Netherlands; ^10^Division of Rheumatology, Immunology, and Allergy; ^11^Division of Genetics, Brigham and Women’s Hospital, Harvard Medical School, Boston, MA 02115, USA; ^12^Program in Medical and Population Genetics, Broad Institute, Cambridge, MA 02142, USA.

Supplementary Table 1. Better fit of time-averaged disease activity on hand SHS than one-time disease activity in the Japanese cohorts

| Dependent  variable | Independent variables | | | | IORRA cohort (n=557) | | KURAMA cohort (n=204) | |
| --- | --- | --- | --- | --- | --- | --- | --- | --- |
|  |  |  |  |  | R^2^ | P value | R^2^ | P value |
| SHS(hand) | RF | D.D. | DAS28(ESR): | Time-averaged DAS28 | 0.254 | 0.001 | 0.463 | 0.054 |
|  |  |  |  | First DAS28 | 0.111 | 1.0 | 0.449 | 0.499 |
|  |  |  |  | Latest DAS28 | 0.188 | 0.153 | 0.457 | 0.176 |
|  |  |  |  | One-time DAS28 (1000 times) | 0.170±0.017 |  | 0.450±0.007 |  |
|  | RF | log(D.D.) | DAS28(ESR): | Time-averaged DAS28 | 0.251 | 0.001 | 0.392 | 0.02 |
|  |  |  |  | First DAS28 | 0.113 | 1.0 | 0.382 | 0.144 |
|  |  |  |  | Latest DAS28 | 0.190 | 0.161 | 0.377 | 0.287 |
|  |  |  |  | One-time DAS28 (1000 times) | 0.172±0.017 |  | 0.373±0.009 |  |
|  | RF | D.D. | DAS28(CRP): | Time-averaged DAS28 | na | na | 0.462 | 0.038 |
|  |  |  |  | First DAS28 | na | na | 0.446 | 0.519 |
|  |  |  |  | Latest DAS28 | na | na | 0.456 | 0.139 |
|  |  |  |  | One-time DAS28 (1000 times) | na |  | 0.447±0.007 |  |
|  | RF | log(D.D.) | DAS28(CRP): | Time-averaged DAS28 | na | na | 0.392 | 0.019 |
|  |  |  |  | First DAS28 | na | na | 0.377 | 0.214 |
|  |  |  |  | Latest DAS28 | na | na | 0.376 | 0.232 |
|  |  |  |  | One-time DAS28 (1000 times) | na |  | 0.370±0.009 |  |
|  | RF | D.D. | CDAI: | Time-averaged CDAI | 0.232 | 0.002 | 0.468 | 0.042 |
|  |  |  |  | First　CDAI | 0.0968 | 0.997 | 0.448 | 0.770 |
|  |  |  |  | Latest CDAI | 0.157 | 0.320 | 0.459 | 0.213 |
|  |  |  |  | One-time CDAI (1000 times) | 0.149±0.022 |  | 0.454±0.007 |  |
|  | RF | log(D.D.) | CDAI: | Time-averaged CDAI | 0.234 | 0.002 | 0.400 | 0.031 |
|  |  |  |  | First CDAI | 0.0992 | 0.997 | 0.382 | 0.420 |
|  |  |  |  | Latest CDAI | 0.159 | 0.578 | 0.381 | 0.463 |
|  |  |  |  | One-time CDAI (1000 times) | 0.151±0.022 |  | 0.381±0.009 |  |
| Log  (SHS hand) | RF | D.D. | DAS28(ESR): | Time-averaged DAS28 | 0.244 | 0.001 | 0.478 | 0.103 |
|  |  |  |  | First DAS28 | 0.102 | 1.0 | 0.453 | 0.952 |
|  |  |  |  | Latest DAS28 | 0.174 | 0.302 | 0.487 | 0.026 |
|  |  |  |  | One-time DAS28 (1000 times) | 0.165±0.016 |  | 0.466±0.009 |  |
|  | RF | log(D.D.) | DAS28(ESR): | Time-averaged DAS28 | 0.242 | 0.001 | 0.47 | 0.021 |
|  |  |  |  | First DAS28 | 0.103 | 1.0 | 0.447 | 0.498 |
|  |  |  |  | Latest DAS28 | 0.175 | 0.316 | 0.465 | 0.052 |
|  |  |  |  | One-time DAS28 (1000 times) | 0.167±0.016 |  | 0.448±0.009 |  |
|  | RF | D.D. | DAS28(CRP): | Time-averaged DAS28 | na | na | 0.464 | 0.109 |
|  |  |  |  | First DAS28 | na | na | 0.450 | 0.881 |
|  |  |  |  | Latest DAS28 | na | na | 0.473 | 0.02 |
|  |  |  |  | One-time DAS28 (1000 times) | na |  | 0.456±0.006 |  |
|  | RF | log(D.D.) | DAS28(CRP): | Time-averaged DAS28 | na | na | 0.454 | 0.033 |
|  |  |  |  | First DAS28 | na | na | 0.438 | 0.389 |
|  |  |  |  | Latest DAS28 | na | na | 0.452 | 0.05 |
|  |  |  |  | One-time DAS28 (1000 times) | na |  | 0.438±0.007 |  |
|  | RF | D.D. | CDAI: | Time-averaged CDAI | 0.200 | 0.001 | 0.467 | 0.074 |
|  |  |  |  | First CDAI | 0.082 | 1.0 | 0.451 | 0.840 |
|  |  |  |  | Latest CDAI | 0.146 | 0.203 | 0.472 | 0.025 |
|  |  |  |  | One-time CDAI (1000 times) | 0.133±0.015 |  | 0.456±0.006 |  |
|  | RF | log(D.D.) | CDAI: | Time-averaged CDAI | 0.202 | 0.001 | 0.459 | 0.047 |
|  |  |  |  | First CDAI | 0.084 | 1.0 | 0.443 | 0.445 |
|  |  |  |  | Latest CDAI | 0.147 | 0.209 | 0.451 | 0.162 |
|  |  |  |  | One-time CDAI (1000 times) | 0.134±0.015 |  | 0.443±0.008 |  |

R^2^= R-squared value, SHS= modified Sharp/van der Heijde score; DAS28= disease activity score 28; CDAI: clinical disease ativity index; D.D.= disease duration, and RF= rheumatoid factor. The data were expressed with mean±standard deviation, and na= not assigned.Supplementary

Supplementary Table 2. Better fit of time-averaged disease activity on hand and foot SHS than one-time disease activity in the KURAMA cohort.

| Dependent  variable | Independent variables | | | | KURAMA cohort (n=204) | |
| --- | --- | --- | --- | --- | --- | --- |
|  |  |  |  |  | R^2^ | P value |
| SHS  (hand & foot) | RF | D.D. | DAS28(ESR): | Time-averaged DAS28 | 0.460 | 0.034 |
|  |  |  |  | First DAS28 | 0.451 | 0.293 |
|  |  |  |  | Latest DAS28 | 0.454 | 0.167 |
|  |  |  |  | One-time DAS28 (1000 times) | 0.449±0.005 |  |
|  | RF | log(D.D.) | DAS28(ESR): | Time-averaged DAS28 | 0.374 | 0.016 |
|  |  |  |  | First DAS28 | 0.366 | 0.091 |
|  |  |  |  | Latest DAS28 | 0.360 | 0.264 |
|  |  |  |  | One-time DAS28 (1000 times) | 0.356±0.007 |  |
|  | RF | D.D. | DAS28(CRP): | Time-averaged DAS28 | 0.459 | 0.027 |
|  |  |  |  | First DAS28 | 0.447 | 0.376 |
|  |  |  |  | Latest DAS28 | 0.453 | 0.127 |
|  |  |  |  | One-time DAS28 (1000 times) | 0.447±0.005 |  |
|  | RF | log(D.D.) | DAS28(CRP): | Time-averaged DAS28 | 0.373 | 0.016 |
|  |  |  |  | First DAS28 | 0.361 | 0.176 |
|  |  |  |  | Latest DAS28 | 0.360 | 0.205 |
|  |  |  |  | One-time DAS28 (1000 times) | 0.355±0.007 |  |
|  | RF | D.D. | CDAI: | Time-averaged CDAI | 0.463 | 0.039 |
|  |  |  |  | First CDAI | 0.448 | 0.731 |
|  |  |  |  | Latest CDAI | 0.454 | 0.276 |
|  |  |  |  | One-time CDAI (1000 times) | 0.452±0.005 |  |
|  | RF | log(D.D.) | CDAI: | Time-averaged CDAI | 0.378 | 0.040 |
|  |  |  |  | First CDAI | 0.363 | 0.426 |
|  |  |  |  | Latest CDAI | 0.361 | 0.514 |
|  |  |  |  | One-time CDAI (1000 times) | 0.362±0.007 |  |
| Log (SHS  hand & foot) | RF | D.D. | DAS28(ESR): | Time-averaged DAS28 | 0.481 | 0.200 |
|  |  |  |  | First DAS28 | 0.465 | 0.937 |
|  |  |  |  | Latest DAS28 | 0.498 | 0.005 |
|  |  |  |  | One-time DAS28 (1000 times) | 0.474±0.007 |  |
|  | RF | log(D.D.) | DAS28(ESR): | Time-averaged DAS28 | 0.473 | 0.042 |
|  |  |  |  | First DAS28 | 0.455 | 0.620 |
|  |  |  |  | Latest DAS28 | 0.479 | 0.002 |
|  |  |  |  | One-time DAS28 (1000 times) | 0.459±0.007 |  |
|  | RF | D.D. | DAS28(CRP): | Time-averaged DAS28 | 0.471 | 0.195 |
|  |  |  |  | First DAS28 | 0.466 | 0.565 |
|  |  |  |  | Latest DAS28 | 0.485 | 0.003 |
|  |  |  |  | One-time DAS28 (1000 times) | 0.468±0.005 |  |
|  | RF | log(D.D.) | DAS28(CRP): | Time-averaged DAS28 | 0.462 | 0.047 |
|  |  |  |  | First DAS28 | 0.451 | 0.454 |
|  |  |  |  | Latest DAS28 | 0.466 | 0.015 |
|  |  |  |  | One-time DAS28 (1000 times) | 0.452±0.005 |  |
|  | RF | D.D. | CDAI: | Time-averaged CDAI | 0.475 | 0.055 |
|  |  |  |  | First CDAI | 0.465 | 0.597 |
|  |  |  |  | Latest CDAI | 0.481 | 0.010 |
|  |  |  |  | One-time CDAI (1000 times) | 0.467±0.004 |  |
|  | RF | log(D.D.) | CDAI: | Time-averaged CDAI | 0.468 | 0.036 |
|  |  |  |  | First CDAI | 0.452 | 0.621 |
|  |  |  |  | Latest CDAI | 0.464 | 0.104 |
|  |  |  |  | One-time CDAI (1000 times) | 0.455±0.006 |  |

R^2^= R-squared value, SHS= modified Sharp/van der Heijde score; DAS28= disease activity score 28; CDAI: clinical disease ativity index; D.D.= disease duration, and RF= rheumatoid factor. The data were expressed with mean±standard deviation, and na= not assigned.

Supplementary Figure 1 Better fit of time-averaged DAS28 on hands and foot SHS in the KURAMA cohort.


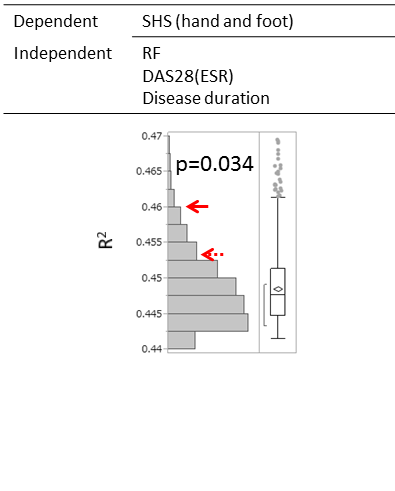


The results of empirical fitting of the DAS28 on hands and foot SHS are indicated in the 204 subjects in the KURAMA cohort. The solid and broken red arrow indicate time-averaged DAS28 and the last visit before X-rays, respectively.

Supplementary Figure 2. A poor model fit using the latest DAS28 within 6 months before joint X-rays in the IORRA cohort.


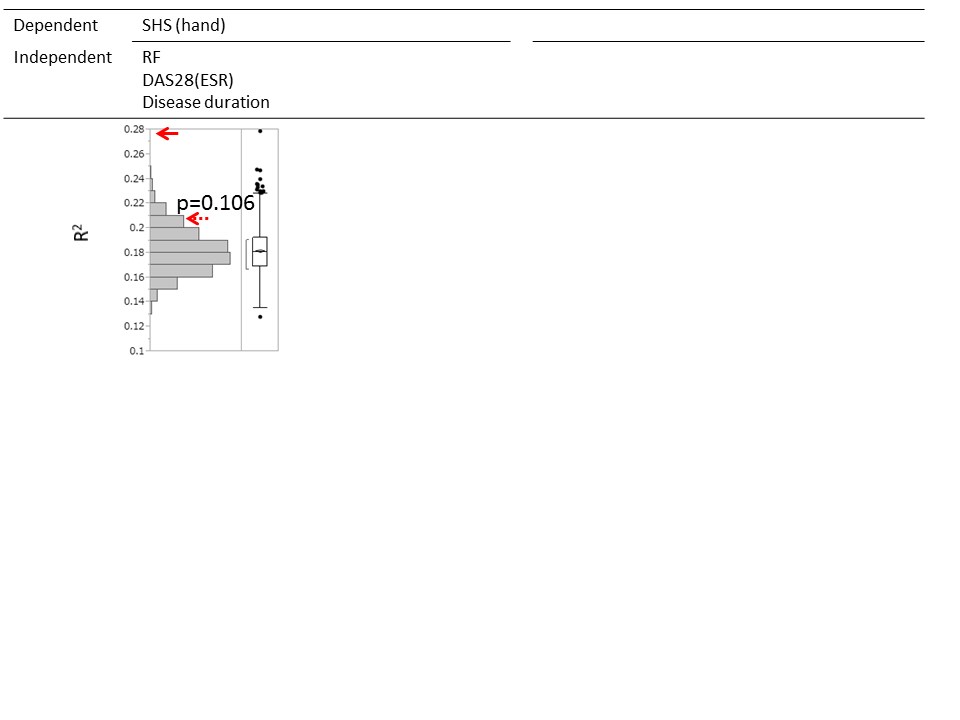


The results of empirical fitting of the DAS28 are indicated in the 516 subjects in the IORRA cohort whose DAS28 within six months before joint X-rays were available. The broken red arrow indicates p-value of the DAS28 evaluated at the time nearest the joint X-rays. The solid red arrow indicates time-averaged DAS28.

Supplementary Figure 3. Good fit of time-averaged DAS28 was driven by subjects with more numbers of DAS28 in each Japanese cohort.


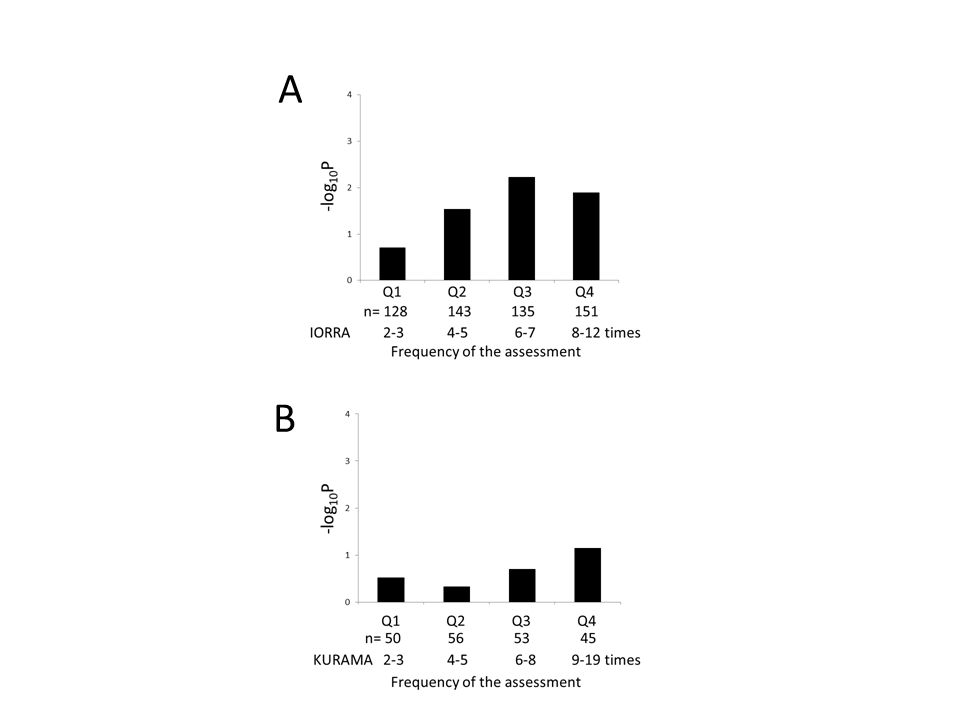


The barplot indicates –log_10_ empirical P-values of R^2^ of time-averaged DAS28 in each quadrant based on the number of time points of DAS28 in A) the IORRA and B) KURAMA cohorts.
